# Rapid engineering of SARS-CoV-2 therapeutic antibodies to increase breadth of neutralization including BQ.1.1, CA.3.1, CH.1.1, XBB.1.16, and XBB.1.5

**DOI:** 10.1093/abt/tbad006

**Published:** 2023-04-13

**Authors:** Kevin C Entzminger, Jonathan K Fleming, Paul D Entzminger, Lisa Yuko Espinosa, Alex Samadi, Yuko Hiramoto, Shigeru C J Okumura, Toshiaki Maruyama

**Affiliations:** Antibody Discovery, Abwiz Bio Inc., San Diego, CA 92121, USA; Antibody Discovery, Abwiz Bio Inc., San Diego, CA 92121, USA; Antibody Discovery, Abwiz Bio Inc., San Diego, CA 92121, USA; Antibody Discovery, Abwiz Bio Inc., San Diego, CA 92121, USA; Antibody Discovery, Abwiz Bio Inc., San Diego, CA 92121, USA; Antibody Discovery, Abwiz Bio Inc., San Diego, CA 92121, USA; Antibody Discovery, Abwiz Bio Inc., San Diego, CA 92121, USA; Antibody Discovery, Abwiz Bio Inc., San Diego, CA 92121, USA

**Keywords:** antibody engineering, neutralizing antibody, XBB.1.5, COVID-19, SARS-CoV-2

## Abstract

SARS-CoV-2 Omicron variant XBB.1.5 has shown extraordinary immune escape even for fully vaccinated individuals. There are currently no approved antibodies that neutralize this variant, and continued emergence of new variants puts immunocompromised and elderly patients at high risk. Rapid and cost-effective development of neutralizing antibodies is urgently needed. Starting with a single parent clone that neutralized the Wuhan-Hu-1 strain, antibody engineering was performed in iterative stages in real time as variants emerged using a proprietary technology called STage-Enhanced Maturation. An antibody panel that broadly neutralizes currently circulating Omicron variants was obtained by in vitro affinity maturation using phage display. The engineered antibodies show potent neutralization of BQ.1.1, XBB.1.16, and XBB.1.5 by surrogate virus neutralization test and pM K_D_ affinity for all variants. Our work not only details novel therapeutic candidates but also validates a unique general strategy to create broadly neutralizing antibodies to current and future SARS-CoV-2 variants.

## Teaser

We report engineering and characterization of SARS-CoV-2 therapeutic antibodies that neutralize all Omicron variants to date. Surrogate virus neutralization assay shows low ng/mL IC_50_ values and biolayer interferometry shows pM affinity. The use of a proprietary platform technology, STage-Enhanced Maturation, is detailed.

## INTRODUCTION

During the 3 years since the outbreak of coronavirus disease 2019 (COVID-19), the severe acute respiratory syndrome coronavirus 2 (SARS-CoV-2) virus has proven exceptionally adept at mutating to evade the immune response [1]. The emergence of the first Omicron variant BA.1, having far more mutations in the spike receptor binding domain (RBD) compared to earlier variants, dramatically increased the susceptibility of even previously vaccinated or infected individuals [2]. Later Omicron sublineages BA.2 (from which XBB.1.5 is derived) and BA.4/5 (from which BQ.1.1 is derived) have continued to evolve additional immune-evading mutations, further increasing the likelihood of breakthrough infections [1, 3]. At the time of writing, March 2023, XBB.1.5 and BQ.1.1 are dominant circulating strains, comprising 90% of all infections (cdc.gov). As a master of immune evasion, XBB.1.5 has been deemed the most transmissible variant yet [4] and has the potential to dominate other variants worldwide.

Antibody therapeutics are the standard of care for at-risk populations who are immunocompromised and thus susceptible to adverse COVID infection. Yet antibody therapeutics approved for treatment of COVID-19 have lost efficacy soon after new strains emerged. BA.1 significantly reduced the potency of first-generation antibody therapeutics such as REGEN-COV (casirivimab–imdevimab) [1]. BQ.1.1 resists neutralization by single or cocktail monoclonal antibody (mAb) therapies including sotrovimab, bebtelovimab, bamlanivimab–etesevimab, and evusheld (cilgavimab–tixagevimab) [5, 6]. XBB.1.5 has been shown to similarly evade neutralizing antibodies [3]. There has been a call for novel, broadly active mAbs urgently needed for prophylactic and/or therapeutic treatment in patients at high risk [5, 7, 8], especially given the danger associated with re-infection [9]. No currently U.S. Food & Drug Administration (FDA) approved antibody therapeutics neutralize the latest Omicron variants BQ.1.1 and XBB.1.5.

Existing strategies to engineer neutralizing therapeutic mAbs typically rely on antibody isolation from infected or vaccinated individuals or from immunized humanized mice [10–12]. However, this process is poorly adapted for rapidly evolving targets such as SARS-CoV-2, where the lead discovery phase must be repeated each time a new variant emerges. Instead, we devised an approach that evolves antibody neutralization breadth in real time as the virus itself evolves. Starting from a humanized lead candidate that showed strong neutralization of the Wuhan-Hu-1 strain, we created six separate complementarity determining region (CDR)-targeted libraries and selected the libraries on Wuhan-Hu-1 spike trimer to create diverse CDR pools. These CDRs were then randomly paired and iteratively selected on SARS-CoV-2 variants as they emerged, resulting in a final panel of seven clones that strongly neutralize all Omicron variants including XBB.1.5 and BQ.1.1. This study not only details potential high-value therapeutic mAbs but also validates a general strategy to elicit broadly neutralizing mAbs for SARS-CoV-2 or other viruses.

## MATERIALS AND METHODS

### Preparation of recombinant proteins

Spike trimers were prepared in-house for use in all assays. SARS-CoV-2 variants were made by overlap PCR and cloned to pCAGGS vector for production. Spike trimers include polybasic cleavage site mutation/deletion and solubilizing mutations K986P/V987P as well as a trimerization motif and polyhistidine tag [13]. Following large-scale purification, 300 μg of deoxyribonucleic acid (DNA) was transfected to HEK293T cells, with media harvested by centrifugation and filtration ⁓7 days post transfection. His-tagged spike trimer was purified using a Nickel Nitriloacetic acid (Ni-NTA) column: the spike trimer was washed with 50 mM imidazole then eluted with 100 mM and 250 mM imidazole, followed by overnight dialysis in DPBS. Quality and purity were assessed by SDS-PAGE (sodium dodecyl sulfate–polyacrylamide gel electrophoresis) gel and activity was assessed by testing for binding to angiotensin converting enzyme 2-human IgG Fc fusion (ACE2-Fc) by ELISA. In-house biotinylated trimers were prepared for biolayer interferometry (BLI) studies using EX-Link™ NHS-LC-Biotin (ThermoFisher A39257) followed by purification with 7 K MWCO Zeba™ Spin Desalting Columns (ThermoFisher 89882). Spike trimers used in the study include Wuhan-Hu-1 (GenBank MN908947; Abwiz Bio Cat. #2720) as well as variants Beta (Abwiz Bio Cat. #2652), Delta (Abwiz Bio Cat. #2611), BA.1 (Abwiz Bio Cat. #2672), BA.2 (Abwiz Bio Cat. #2460), BA.2.75 (Abwiz Bio Cat. #2664), BA.2.75.2 (Abwiz Bio Cat. #2676), BA.2.3.20 (Abwiz Bio Cat. #2660), BN.1 (Abwiz Bio Cat. #2696), XBB (Abwiz Bio Cat. #2700), XBB.1/XBB.1.9 (Abwiz Bio Cat. #2648), XBB.1.5/XBB.1.9.1 (Abwiz Bio Cat. #2712), XBB.1.16 (Abwiz Bio Cat. #2747), BA.4.6 (Abwiz Bio Cat. #2692), BA.5 (Abwiz Bio Cat. #2688), BQ.1 (Abwiz Bio Cat. #2704), BQ.1.1 (Abwiz Bio Cat. #2668), BF.7/BA.5.2.6/BF.11 (Abwiz Bio Cat. #2708), CH.1.1 (Abwiz Bio Cat. #2724), CA.3.1 (Abwiz Bio Cat. #2728), and BR.2.1 (Abwiz Bio Cat. #2736).

### Immunization of rabbits

Three New Zealand White rabbits were immunized with a recombinant protein including the receptor-binding domain (RBD) of SARS-CoV-2 (Wuhan-Hu-1 strain). The immune sera were tested by ELISA using spike trimer, and the rabbit that showed the best titer was selected for library construction.

### Library construction

The bone marrow and spleen cells were collected and homogenized in TRI reagent (Molecular Research Center). Total ribonucleic acid (RNA) was isolated from the homogenate according to the manufacturer’s protocol. Messenger RNA was purified using messenger ribonucleic acid (mRNA) purification kit (Macherey-Nagel) according to the manufacturer’s protocol. First strand complementary deoxyribonucleic acid (cDNA) was synthesized using PowerScribe MMLV Reverse Transcriptase (Monserate Biotechnology Group). cDNA was engineered using a method described in the US patent 9,890,414 and amplified with a single non-gene-specific primer. Amplified products were purified, digested, and sequentially ligated into a proprietary rabbit Fab phagemid vector.

### Phage panning

Library DNA was transformed into XL1-Blue cells (Agilent) for overnight growth and phage production following addition of M13KO7 Helper Phage (New England Biolabs). Phage were precipitated the following day from culture supernatant by addition of 4% PEG-8000/3% NaCl on ice, followed by pelleting and resuspension in 1% BSA/Dulbecco’s phosphate-buffered saline (DPBS) (blocking buffer). High-bind microtiter wells (Immulon 4HBX) were coated with recombinant spike trimer overnight, washed with PBS, and blocked with blocking buffer for 1 h at 37°C. Phage were added for incubation at 37°C for 1 h, followed by washing with PBS, elution with 0.1 M HCl, and neutralization with 2 M Tris. Neutralized phage were used to infect ER2738 (New England Biolabs) cells for overnight propagation and precipitation as described above. Panning was performed for four or more rounds, with increasing washing stringency in later rounds to enrich specific binders. During phage panning of STage-Enhanced Maturation (STEM) libraries, [1] the library was first transiently heated to 65°C then cooled to improve thermoresistance as described previously [14], followed by [2] subtraction of the library on baculovirus particle (BVP) coated microtiter wells to remove polyreactive clones before selection on spike protein trimer.

### Fab screening

Following the final round of phage panning, single colonies were prepared from the eluted page. Colonies were picked to 96-well culture plates with 1 mL of Super Broth medium and 50 μg/mL of carbenicillin. The culture plates were incubated at 37°C and 1 mM Isopropyl ß-D-1-thiogalactopyranoside (IPTG) was added to induce overnight soluble Fab production at 30°C. For Fab ELISA, microtiter wells were coated with 100 μL of spike trimer at 1–2 μg/mL in PBS. Following washing and blocking, culture supernatants containing soluble Fab were added to the wells for incubation at 37°C for 1 h. After washing, bound Fab was detected with peroxidase conjugated goat anti-rabbit IgG F(ab’)_2_ (Thermo Fisher Scientific) or anti-human IgG F(ab’)_2_ secondary antibodies (Jackson ImmunoResearch). Positive clones were Polymerase Chain Reaction (PCR) amplified and sequenced by Sanger sequencing (Eton Biosciences).

### IgG production and humanization of rabbit antibody

Light and heavy chains of rabbit clone C-A11 were PCR amplified, digested, and cloned into a bi-cistronic rabbit IgG vector for expression. All antibodies and recombinant ACE2-Fc (Abwiz Bio Cat. #2566) were produced by transient transfection of HEK293T cells with media harvested by centrifugation and filtration ⁓7 days post transfection. IgGs were purified by protein A column (Cytiva) followed by overnight dialysis in DPBS. CDRs of C-A11 rabbit antibody were grafted into selected human germline genes (IGHV3-23*04 and IGKV-39*01) to create a humanized clone hN2Y. An expanded structural definition of CDRs was used, based on observed diversity from an internal database of rabbit antibodies. Following gene synthesis, hN2Y light and heavy chain genes were cloned into a bi-cistronic human IgG vector for production as above. S728-1157 light and heavy chain genes were synthesized by Twist Bioscience and cloned into our IgG expression vector, CR3022 expression plasmids were obtained from BEI Resources, NIAID, NIH for expression and purification as described above.

### Surrogate virus neutralization test of purified IgG

Surrogate virus neutralization tests (sVNTs) were performed according to the protocol described previously [15] with some modifications. Microtiter wells were coated with 100 μL of ACE2-Fc (Abwiz Cat. #2566) at 2 μg/mL in PBS and blocked with 200 μL of 1% BSA/PBS. Purified IgG were mixed 1:1 with a recombinant spike trimer at RT and added to the wells. The bound spike trimer was detected with peroxidase conjugated anti-his tag antibody (Jackson ImmunoResearch 300-035-240). All assays were performed in triplicate. Raw ELISA values were normalized to six replicate positive wells omitting IgG and to three negative wells omitting both IgG and spike trimer, with negative values forced to zero. Graphpad Prism was used to prepare graphs and to derive inhibition of spike trimer binding to ACE2 half maximal inhibitory concentration (IC_50_) values by fitting to a four-parameter logistic curve. Bebtelovimab, tixagevimab, cilgavimab, and S2X324 were purchased from Proteogenix.

### STage-Enhanced Maturation antibody engineering

In Stage 1, Six single CDR mutant libraries were designed based on amino acid usage by structurally characterized human antibodies sharing similar canonical CDR structures among a subset that possessed identical germline genes. This design strategy further humanizes the amino acid usage in the CDRs and helps to avoid unwanted mutations that could negatively affect the structure and folding of the antibodies while constraining library size to a diversity that could be sampled using our phage libraries. Mutagenic oligos were synthesized and used to construct separate CDR libraries by fragment amplification and overlap PCR using hN2Y gene as a template. Following digestion, ligation was performed at large scale using 10 μg digested vector DNA. These single CDR mutant libraries were panned on Wuhan-Hu-1 spike trimer to remove non-productive clones and to enrich all possible CDR mutants that retain binding to the spike trimer. Phage panning and Fab screening was performed as described above.

In Stage 2, round four output phage from Stage 1 were used to amplify targeted CDR regions and combined by overlap PCR into separate light chain (Lx; paired with the wild type heavy chain) or heavy chain (Hx; paired with the wild type light chain) libraries. The Lx library was panned on the Beta spike trimer, and the Hx library was panned separately on Wuhan-Hu-1, Alpha, Beta, and Epsilon spike trimers. Phage panning and Fab screening was performed as described above. LxC1-G10 clone was identified from the Lx library and was cloned to IgG format and characterized as described above.

In Stage 3, round four output phage from Stage 2 were used to amplify light and heavy chain libraries for sequential cloning and pairing to create a single combined library. This library was panned for six rounds on BA.5 followed by two rounds on BA.2.75 and one round on either BQ.1.1 or XBB spike trimers. Fabs were screened similarly as described above and tested for binding to the respective spike trimer used for selection. Phage panning and Fab screening was performed as described above. Clones possessing 6R8 or 6R9 prefixes were identified from this screening and were converted to IgG format for characterization as described above.

**Figure 1:**
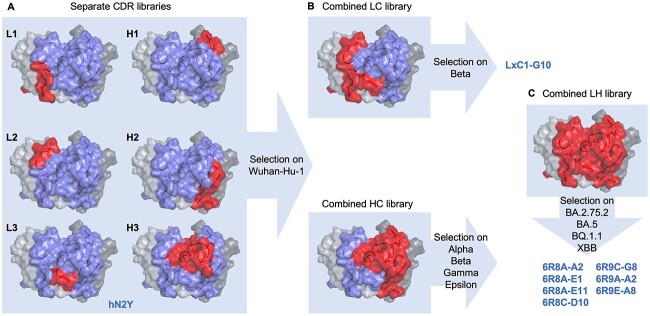
STEM antibody engineering strategy. (A) Rabbit immunization with Wuhan-Hu-1 SARS-CoV-2 RBD was paired with phage display to identify a potent neutralizing clone; this mAb was humanized to create the lead candidate hN2Y. Single CDR libraries were constructed and selected on Wuhan-Hu-1 spike trimer to create a pool of functional CDR sequences. hN2Y predicted structure is shown, with all CDRs colored blue and targeted CDR(s) for a given library colored red. (B) Pre-selected CDR pools were amplified from phage and combined by overlap PCR to create separate combined light and combined heavy chain libraries. The light chain library was further selected on the Beta variant, resulting in the engineered clone LxC1-G10, which showed potent neutralization of BA.1 and was thoroughly characterized by the CoVIC at La Jolla Institute of Immunology [25]. The heavy chain library was selected on a combination of Alpha, Beta, Gamma, and Epsilon variants to broaden neutralization activity. (C) Pre-selected light and heavy chain libraries were then paired to create a single combined library. This library was selected on late-stage Omicron variants including BA.2.75.2, BA.5, BQ.1.1, and XBB to create broadly neutralizing 6R8/6R9 series antibodies.

### Biolayer interferometry

Biotinylated spike trimers were prepared in-house as described above except BA.1 (ACROBiosystems SPN-C82Ee). BLI was performed using Octet Red96e with data collection at 30°C and sample shaking at 1000 rpm during all steps. All IgGs and spike trimers were prepared in running buffer containing 2% BSA, 0.002% Tween-20, PBS, pH 7.4. Following 4 min baseline, 3 μg/mL of biotinylated trimer was captured to streptavidin sensors (Sartorius 18-5019) for 4 min followed by an additional 4 min baseline. IgG binding was monitored for 4 min followed by 30 min dissociation in fresh buffer only wells. Each IgG was tested in 1:2 titration series from 10 to 0.16 nM. A reference sensor omitting IgG was included in each assay and used for subtraction. All data were globally fit to 1:1 binding model to derive kinetic and affinity values, only omitting individual traces or times due to sensor error or low signal. For the in-tandem epitope binning assay, following the same baseline and antigen loading steps described above, an initial 10 min 10 nM IgG-binding step was performed to allow for signal saturation, followed by immediate transfer to wells containing 10 nM IgGs to monitor binding for an additional 10 min. The final nm shift values after the second IgG incubation step were used to prepare a heatmap of results.

## RESULTS

### Therapeutic mAbs were engineered by STEM in real time as SARS-CoV-2 variants emerged

Soon after the onset of the COVID-19 pandemic, we used our rabbit discovery platform, which pairs rabbit immunization with Fab-phage display, to create a therapeutic lead candidate mAb. Rabbits were immunized with Wuhan-Hu-1 RBD containing protein, and phage libraries were selected on Wuhan-Hu-1 spike protein trimer. Fab neutralization assay was used to identify the potent clone C-A11. We then grafted CDRs onto a human framework to create the humanized clone hN2Y, which strongly neutralized the Wuhan-Hu-1 strain. As evasive SARS-CoV-2 variants emerged, many groups attempted to identify novel antibodies from vaccinated or infected patient samples [16, 17]. We instead employed our STEM platform to broaden neutralization potency of hN2Y to SARS-CoV-2 variants as they emerged in real time (Fig. 1).

**Figure 2:**
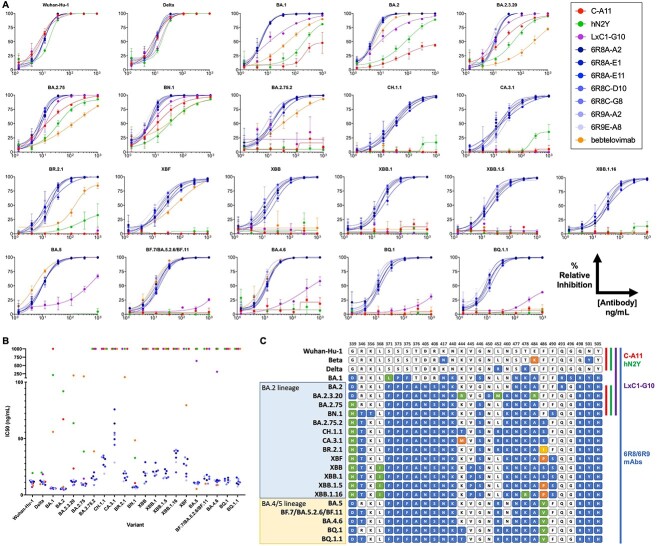
Broad SARS-CoV-2 neutralization for engineered mAbs by sVNT. (A) Purified mAbs were tested by sVNT [15]. Wells were coated with recombinant ACE2-Fc, and His-tagged spike trimer protein was mixed with serially diluted mAb prior to addition. Bound spike trimer was detected by anti-His tag antibody. All experiments were performed in triplicates. 6R8/6R9 clones (blue) showed highly potent and broad neutralization of all Omicron variants tested including XBB.1.5 and BQ.1.1. LxC1-G10 (purple) light chain engineered clone showed improved binding compared to lead humanized candidate hN2Y (green) or parental rabbit clone C-A11 (red), though it lost potency for later Omicron variants. Bebtelovimab (orange) showed no neutralization of later Omicron variants. (B) IC_50_ values for all variants, with non-neutralizing clones reported at 1000 ng/mL. (C) RBD residues modified in the different SARS-CoV-2 variants compared to the Wuhan-Hu-1 strain. Residues are colored by mutation. Colored side bars for each mAb note variants that are neutralized with IC_50_ <100 ng/mL.

Single CDR mutant libraries of hN2Y were first created (Fig. 1A). CDR libraries were designed based on observed amino acid frequencies for characterized human antibodies possessing a similar germline and predicted CDR canonical structure usage to both further humanize CDRs and to avoid introduction of sequence liabilities. By using phage display, each CDR library can be sampled at sizes up to 2E + 10. These separate CDR libraries were first selected on Wuhan-Hu-1 spike trimer to eliminate non-binders and to isolate a large pool of diverse clones. Then, CDR pools from the selected phage populations were amplified and randomly paired by overlap PCR to create secondary combined light chain and combined heavy chain libraries (Fig. 1B). To increase neutralization breadth, the combined light chain library was selected on the Beta variant, and the combined heavy chain library was selected separately on Alpha, Beta, Gamma, and Epsilon variants. We identified an exceptional candidate from the combined light chain library, LxC1-G10, which showed good neutralization of BA.1 and BA.2. After the emergence of later BA.2 lineage Omicron variants, we again used our platform to further improve potency. Light and heavy chains were separately amplified from the pre-selected phage pools and further combined to create a single library (Fig. 1C). The library was selected on BA.5, BA.2.75.2, BQ.1.1, and XBB variants, all of which possess mutation at F486. A diverse set of seven highly potent, broadly neutralizing IgGs (6R8/6R9 clones) was identified following eight or nine rounds of panning and is characterized below.

### In vitro neutralization assay shows broadly neutralizing mAbs

Neutralization titers for sera or mAbs measured using sVNT, which assays blocking of recombinant spike trimer to recombinant ACE2 protein by ELISA, shows strong correlation with results from pseudovirus or live virus neutralization tests [15]. We thus used high-throughput sVNT to quantify neutralization potency of mAbs across SARS-CoV-2 variants. In this assay, recombinant human Fcγ-tagged ACE2 receptor is immobilized while IgGs are pre-incubated with spike trimer before addition to the blocked ACE2 coated wells. ACE2-bound spike trimer is detected by affinity tag using ELISA, and IC_50_ values are derived. mAbs tested include the original neutralizing rabbit clone C-A11, the parent humanized clone hN2Y, LxC1-G10 possessing mutations across light chain CDRs, 6R8/6R9 clones possessing mutations across both heavy and light chain CDRs, and therapeutic mAb bebtelovimab.

Notably, humanization of the rabbit antibody did not significantly reduce efficacy, as hN2Y retained similar neutralization potency for Wuhan-Hu-1 and Delta variants compared to C-A11 (Fig. 2A and B and Table 1). However, both IgGs showed significantly reduced neutralization of the original Omicron variant BA.1. LxC1-G10 contains mutations within all three light chain CDRs compared to hN2Y. Neutralization of early Omicron variants by LxC1-G10 was significantly improved to low ng/mL IC_50_ values for BA.1, BA.2, BA.2.3.20, BA2.75, and BN.1. However, all three mAbs lost efficacy against variant BA.2.75.2, which is notably different compared to these others due to F486S (Fig. 2C). LxC1-G10 showed effective neutralization of BA.2.12.1 lacking F486 mutation but weak neutralization of BA.4/5 (possessing F486V) in a pseudovirus neutralization test by CoVIC at La Jolla Institute [18] in concordance with our observation in sVNT.

**Table 1:** IC_50_ values derived from sVNT data. Calculated IC_50_ values from sVNT assay are provided including 95% confidence intervals. 6R8/6R9 clones show low ng/mL IC_50_ values for all SARS-CoV-2 variants tested. NA, no significant neutralization activity (>1000 ng/mL); UD, undefined

**IC50 (ng/mL)**	**Wuhan-Hu-1**	**Delta**	**BA.1**	**BA.2**	**BA.2.3.20**	**BA.2.75**	**BN.1**	**CH.1.1**	**BA.2.75.2**	**CA.3.1**	**BR.2.1**	**XBF**	**XBB**	**XBB.1.5/XBB.1.9.1**	**XBB.1/XBB.1.9**	**XBB.1.16**	**BA.5**	**BF.7/BA.5.2.6/BF.11**	**BA.4.6**	**BQ.1**	**BQ.1.1**
**C-A11**	10.1	11.6	NA	67.1	23.4	10.2	10.5	NA	NA	NA	NA	NA	NA	NA	NA	NA	NA	NA	NA	NA	NA
**hN2Y**	19.4	19.8	211	91.9	63.6	38.5	32.8	NA	NA	NA	NA	NA	NA	NA	NA	NA	NA	NA	NA	NA	NA
**LxC1-G10**	7.0	18.3	5.7	4.6	9.1	14.4	7.1	NA	NA	NA	NA	NA	NA	NA	NA	NA	636	NA	309.7	NA	NA
**6R8A-A2**	11.7	12.7	6.2	5.5	11.5	9.2	6.7	31.8	16.5	15.4	18.7	31.8	17.1	19.0	20.9	37.4	10.1	13.1	12.4	15.2	10.3
**6R8A-E1**	11.9	8.9	5.4	5.2	11.5	8.9	6.9	26.6	14.9	13.3	12.6	18.8	20.3	22.8	20.6	33.1	10.9	14.4	12.6	13.2	9.3
**6R8A-E11**	11.4	11.5	5.7	6.2	12.5	9.8	7.6	33.1	20.0	17.9	15.4	27.5	29.0	21.2	28.5	39.3	10.9	17.8	13.3	14.3	15.4
**6R8C-D10**	12.7	11.6	5.8	5.0	12.3	8.1	6.6	25.1	13.3	19.5	19.1	16.3	11.7	14.9	16.9	20.2	10.2	14.1	12.7	11.0	12.0
**6R8C-G8**	12.0	9.7	6.0	5.6	11.6	8.8	7.2	34.9	14.0	15.8	15.9	26.1	19.0	15.7	21.7	39.3	11.5	16.2	11.2	12.5	11.1
**6R9A-A2**	9.8	10.5	6.7	6.2	8.5	10.5	7.3	33.5	8.7	24.9	20.9	33.3	10.1	14.7	17.8	39.7	9.4	10.0	8.6	9.3	14.1
**6R9E-A8**	13.1	12.6	5.6	5.6	13.5	8.9	5.6	18.8	13.9	17.9	11.8	12.8	19.0	15.9	19.6	28.5	9.8	16.5	12.9	12.5	7.4
**bebtelovimab**	8.1	9.5	55.9	7.2	407	171	48.4	NA	38.6	NA	149	79.5	NA	NA	NA	NA	5.3	9.9	8.8	NA	NA

To recover efficacy, as described above, we engineered a panel of seven mAbs (6R8A-A2, 6R8A-E1, 6R8A-E11, 6R8C-D10, 6R8C-G8, 6R9A-A2, and 6R9E-A8), which show strong neutralization of all Omicron variants to date including BA.1, BA.2, BA.2.3.20, BA.2.75, BN.1, BA.2.75.2, CH.1.1, CA.3.1, BR.2.1, XBF, XBB, XBB.1 (XBB.1.9), XBB.1.5 (XBB.1.9.1), XBB.1.16, BA.5, BF.7 (BA.5.2.6/BF.11), BA.4.6, BQ.1, and BQ.1.1. To our knowledge, these are the most broadly neutralizing ACE2-blocking antibodies characterized to date against the latest Omicron variants of SARS-CoV-2. Compared to both hN2Y and LxC1-G10, these IgG clones possess mutations across CDRs L1, L2, L3, H1, and H3.

Bebtelovimab is a therapeutic SARS-CoV-2 antibody developed by Eli Lilly and first given Emergency Use Authorization by the US FDA in February 2022, though the agency revoked authorization in late November 2022 due to an inability to neutralize variants BQ.1 and BQ.1.1. Bebtelovimab showed no efficacy against these variants by sVNT or against CH.1.1, CA.3.1, XBB, XBB.1, XBB.1.16, or XBB.1.5 (Fig. 2A and B), matching expected results. S728-1157 clone was developed as a broadly neutralizing antibody against Delta and BA.1 [19]. S728-1157 additionally neutralized BA.2, BN.1, and BA.5 by sVNT but failed to neutralize BA.2.75.2, CH.1.1, CA.3.1, BR.2.1, XBF, XBB, XBB.1.5, XBB.1.16, BQ.1, or BQ.1.1 (Fig. S1). Another previously characterized, broadly neutralizing antibody S2X324, identified from human plasma [17, 20], showed strong neutralization of Wuhan-Hu-1, Delta, BA.2, BA.2.75, BR.2.1, XBF, and BA.5 in our sVNT but failed to neutralize CH.1.1, CA.3.1, XBB.1.5, XBB.1.16, BQ.1, or BQ.1.1 (Fig. S2). In contrast, our engineered IgGs neutralized all SARS-CoV-2 variants tested and all currently circulating globally dominant variants to date.

**Figure 3:**
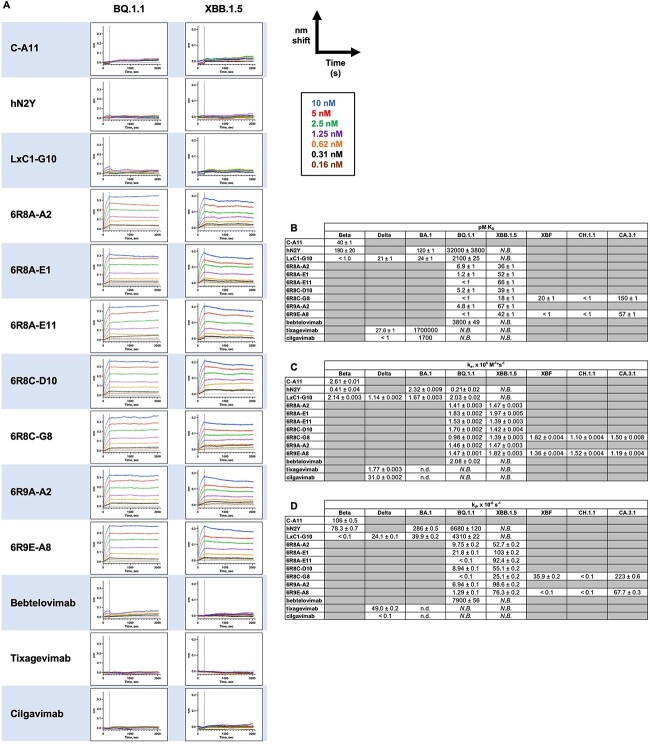
BLI shows high affinity to all SARS-CoV-2 variants for engineered mAbs. In each assay, biotinylated spike protein trimer was captured to the streptavidin-coated sensor surface and dipped into a 7-fold dilution series of IgG at a starting concentration of 10 nM. Binding was monitored for 4 min, followed by dissociation monitored for 30 min. Traces were referenced to a sensor omitting IgG. (A) For each variant tested, each experiment is shown with the same scale y-axis. C-A11, hN2Y, and LxC1-G10 lost binding activity for BQ.1.1 and XBB.1.5, but all seven 6R8/6R9 clones showed strong binding. Therapeutic mAbs tested under the same conditions showed weak or no binding. (B) BLI traces were globally fit to a 1:1 binding model to derive K_D_ affinity values (pM), (C) k_a_, on rates (× 10^6^ M^−1^*s^−1^), and (D) k_d_, off rates (×10^−6^ s^−1^). Some mAb affinities were below the detection limit of the assay (<1 pM) due to exceptionally slow off rates even when measured for 30 min. For some weakly binding clones, affinity, and/or kinetic values could not be determined. *N.B.*, non-binding; n.d., not derived.

### Binding affinity correlates with neutralization potency

BLI was performed by capturing biotinylated spike trimer to a streptavidin-coated sensor surface to monitor binding of serially diluted mAbs, with traces globally fit to 1:1 binding models to derive kinetic and affinity values. hN2Y showed slightly reduced affinity for the Beta variant compared to C-A11 (Fig. S3), but affinity was increased to low pM binding K_D_ for LxC1-G10. LxC1-G10 possessed 24 pM K_D_ affinity for BA.1, 5-fold improved over hN2Y. However, LxC1-G10 binding affinity was itself decreased nearly 100-fold for BQ.1.1 (2.1 nM) compared to BA.1 (24 pM) (Fig. 3A and B).

The seven 6R8/6R9 mAbs possessed 7 pM or lower K_D_ for BQ.1.1 and below 70 pM K_D_ for XBB.1.5. Binding affinity for these mAbs was increased nearly 10 000-fold (for BQ.1.1) or above undetectable levels (for XBB.1.5) compared to parent clone hN2Y. While on-rates were similar (Fig. 3C), the difference in binding affinity for BQ.1.1 is primarily due to a nearly 1000-fold reduction in off-rate, with hN2Y possessing 10^−3^ s^−1^ off-rate while 6R8/6R9 clones showed 10^−6^ s^−1^ off rates (Fig. 3D). 6R8C-G8 and 6R9E-A8 were also tested on XBF, CH.1.1, and CA.3.1 variants; these mAbs showed strong binding to all three, though weaker relative binding to CA.3.1.

Bebtelovimab showed weak binding to BQ.1.1 by BLI and no binding at all to XBB.1.5. Evusheld is an antibody cocktail of Tixagevimab and Cilgavimab first given Emergency Use Authorization by the US FDA in December 2021, though the agency revoked authorization January 2023 due to the inability to neutralize the latest Omicron variants. Both tixagevimab and cilgavimab showed no binding to BQ.1.1 or to XBB.1.5 (Fig. 3A and B). In contrast, our engineered IgGs show low pM binding K_D_ for these dominant SARS-CoV-2 Omicron variants currently circulating in the US.

### Engineered mAbs recognize the same epitope as the parent antibody hN2Y

**Figure 4:**
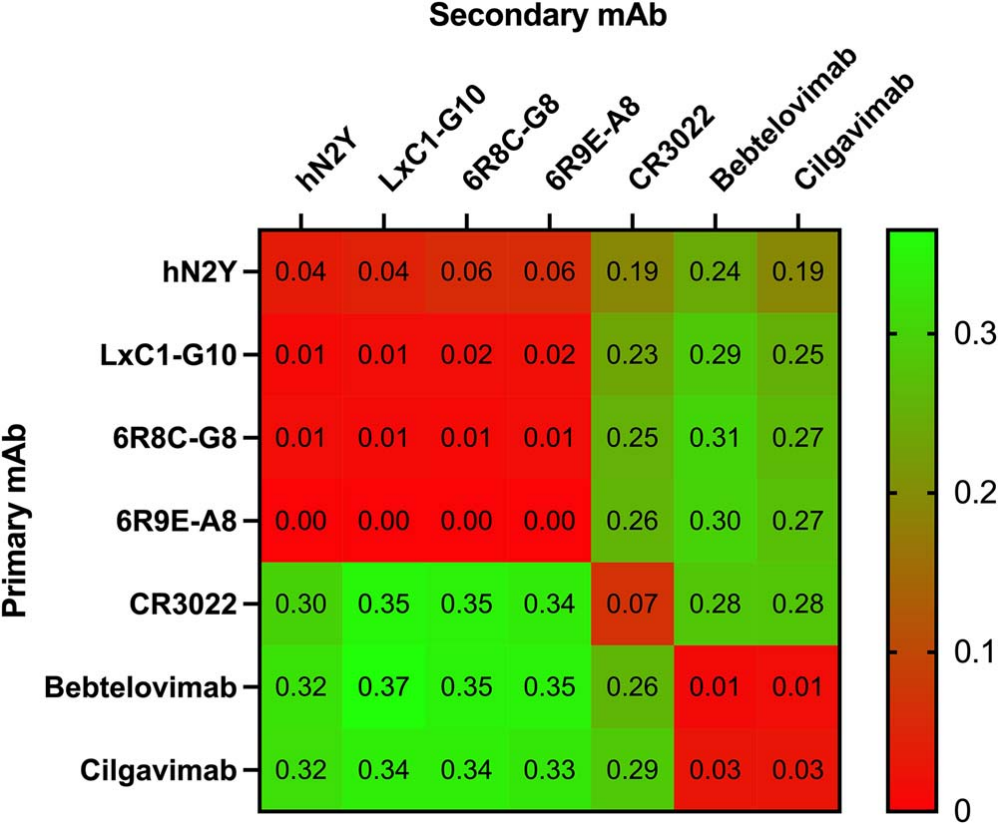
BLI epitope binning shows no change in binding site for engineered mAbs. In an in-tandem assay, 10 nM of primary mAb was first saturated on the spike protein trimer for 10 min, followed by dipping into fresh 10 nM solution of secondary mAb. The nm shift values obtained at the end of the secondary mAb incubation are reported and colored by heatmap. hN2Y, LxC1-G10, 6R8C-G8, and 6R9E-A8 all binned into the same cluster. CR3022 did not cross-compete with any other mAb as expected, while Bebtelovimab and Cilgavimab clustered together.

We then tested whether broader neutralization potency was achieved by significantly altering the epitope. BLI was used for in-tandem epitope binning. In total, 10 nM of IgG was first saturated on the sensor for 600 s, followed by dipping the sensor into new solutions of 10 nM IgGs. The results are presented as a heatmap using the final nm shift values obtained after incubation with the second IgG (Fig. 4). hN2Y, LxC1-G10, 6R8C-G8, and 6R9E-A8 all competed with each other, suggesting that they bind to identical or overlapping epitopes. CR3022, a neutralizing antibody that does not block ACE2 receptor binding [21], does not cross-compete with any antibody as expected. Interestingly, both Bebtelovimab and Cilgavimab, which block ACE2 receptor binding but do not bind BQ.1.1 or XBB.1.5, were binned into a distinct cluster that does not compete with our engineered mAbs. This demonstrates that broader neutralization activity of the engineered IgGs was likely achieved by optimizing binding to conserved SARS-CoV-2 residues within the same epitope as the parent mAb, rather than by significantly changing the binding site.

## DISCUSSION

We have detailed the engineering and activity of broadly neutralizing mAbs that show potent efficacy against all dominant Omicron circulating variants including XBB.1.5 and BQ.1.1. Uniquely, instead of identifying a novel clone from infected or immunized samples, we rather iteratively engineered a single IgG, originally developed for neutralization of the Wuhan-Hu-1 strain.

Our humanized clone hN2Y was derived from a rabbit lead candidate. Rabbits are an excellent source of potent antibodies due to the exceptionally high diversity of their immune repertoire by use of somatic gene conversion and somatic hypermutation [22]. C-A11 rabbit mAb possesses a 19 amino acid CDR H3 and 9 amino acid CDR L3. Long CDR H3 loops, typically defined as ≥24 amino acids, are a common feature of broadly neutralizing antiviral mAbs [23]. Though not technically classified as ‘long,’ the longer CDR H3 may have contributed to C-A11’s potent neutralization of early variants through Delta. Despite high CDR diversity, humanization of rabbit clones is relatively straightforward due to their restricted use of a limited number of germline genes [24]. After humanization, hN2Y showed almost no change in affinity or neutralization activity compared to C-A11 and showed even stronger neutralization of some variants including BA.1 and BA.2.

Omicron variant BA.1 reduced the neutralization potency of hN2Y, as observed for many other therapeutic antibodies developed prior to December 2021. As detailed above, LxC1-G10 was selected by panning a combined light chain library on the Beta variant, which possesses RBD mutations K417N/E484K/N501Y (Fig. 2C). LxC1-G10 showed sub-pM affinity for the Beta variant, >100-fold increased compared to hN2Y (Fig. 3B). This increased binding was sufficient to improve neutralization of early Omicron subvariants BA.1, BA.2, BA.2.3.20, BA.2.75, and BN.1, despite those variants possessing many additional mutations within the spike protein. LxC1-G10 shares an identical heavy chain as hN2Y but possesses over 14 amino acid differences distributed across all light chain CDRs. Light chain CDR mutations alone were thus sufficient to confer significant improvement in binding affinity and neutralization potency.

However, LxC1-G10 lost activity against all Omicron variants possessing a mutation at F486. F486 mutations are found in all later Omicron variants and have been linked to immune evasion [1]. The further engineered 6R8/6R9 series mAbs, selected from panning a combined light and heavy chain library on Omicron variants possessing F486S (BA.2.75.2 and XBB) and F486V (BA.5 and BQ.1.1) mutations, showed exceptional neutralization potency and binding for all SARS-CoV-2 variants tested, including those with alternate amino acids at this site such as F486I (BR.2.1) and F486P (XBF and XBB.1.5). Compared to both hN2Y and LxC1-G10, 6R8/6R9 mAbs possess two mutations in CDR H3 (which is identical among all 6R8/6R9 clones) and 6–9 mutations in CDR H1. Compared to LxC1-G10, 6R8/6R9 mAbs possess 11–13 mutations across all light chain CDRs. Extensive mutagenesis across all CDRs except H2 was thus required to confer the increased neutralization activity and binding affinity observed.

Both our early lead candidate hN2Y (CoVIC-359), and LxC1-G10 (CoVIC-362) were submitted to the CoVIC at La Jolla Institute of Immunology (https://covicdb.lji.org/) for analysis and testing against over 350 other therapeutic antibody candidates [18, 25]. CoVIC is an international effort to conduct side-by-side analyses of candidate antibody therapeutics targeting the SARS-CoV-2 spike protein in standardized assays. Both antibodies showed nearly 100% protection against cell infection in pseudovirus neutralization assay for Beta, Delta, BA.1, BA.1.1, and BA.2 variants, and showed efficacy with live Wuhan-Hu-1 virus in cell-based neutralization assay and during *in vivo* mouse challenge [25]. Both were binned into the same epitope class, RBD-2a, characterized by bivalent intra-spike binding, where both arms of the IgG bind to two spike units within a single trimer. This binding motif was further validated by cryo-EM in the CoVIC study, in which LxC1-G10 was observed to bind to both spike units in the ‘up’ conformation, a conformational state that exposes the ACE2 receptor-binding site [25]. Our epitope binning assay (Fig. 4) demonstrated that the engineered 6R8/6R9 mAbs likely bind in the same RBD-2a epitope class. The increased avidity conferred by bivalent binding likely contributes to the strong neutralization activity and high affinity for 6R8/6R9 mAbs.

In traditional humanization, animal-derived CDRs are grafted into human framework germline genes. Then, further engineering is performed using various methods to obtain the best lead candidate for therapeutic mAb development [26–32]. These methods suffer from notable setbacks including [1] the inability to easily sample mutations that work cooperatively within or across CDRs such as in saturation mutagenesis and CDR walking, [2] introduction of unwanted framework mutations such as in error prone PCR, [3] lack of secondary selection pressure when combining beneficial mutations such as in parallel CDR optimization [26], and [4] almost all omit selection pressure for well-behaved clones. In contrast, our *in vitro* STEM platform is designed to sample the broadest sequence diversity including inter-CDR cooperative mutations and directly incorporates developability filters early during phage panning.

Comparing hN2Y to 6R8/6R9 mAbs, there are four to five amino acid differences in CDR L1, four in CDR L2, four in CDR L3, six to nine in CDR H1, and two in CDR H3, totaling 21–24 amino acid mutations accumulated during affinity maturation. This is a very large number of changes which would be difficult to impossible to obtain by any other established affinity maturation method. Notably, none of the CDR sequences from the 6R8/6R9 clones were identified during screening of single CDR libraries (panned on Wuhan-Hu-1) or separate combined CDR libraries (separately panned on Alpha, Beta, Gamma, Delta, or Epsilon), making it unlikely that a CDR walking [33] or parallel CDR optimization [26] approach would be successful. Alternative methods combine saturated mutagenesis libraries by DNA shuffling [34], but the likelihood of these methods resulting in two or more tandem mutations within an individual CDR, as observed in all 6R8/6R9 clones, is very low due to the improbability of recombination within such close proximity. Computational methods used for affinity maturation have shown promise, but the quality of prediction begins to fall and the complexity in modeling begins to increase dramatically beyond five amino acid mutations, making it impossible to predict the final 6R8/6R9 sequences *in silico* [35]. Taken together, only by applying our STEM platform, which samples exceptionally large diversity across all CDRs, could these potent neutralizing antibodies be created. The absence of competitor mAbs showing similar potency further underscores our approach.

We have characterized an antibody panel that outperforms all existing FDA approved antibody therapeutics against all globally dominant circulating SARS-CoV-2 strains to date. This study validates our unique STEM platform. Though our lead humanized clone showed no binding to or neutralization of XBB.1.5, we successfully recovered potency by engineering through repeated sampling and combination of CDRs progressively against SARS-CoV-2 variants. The ability to recover neutralization activity from a single lead mAb demonstrates the exceptional CDR sequence diversity that can be obtained by applying an iterative selection and library creation strategy. By continuing to engineer a single mAb, lead time was significantly reduced and significant resources were saved by avoiding the need to repeat the discovery process by immunization or screening. *In vitro* affinity maturation of a single neutralizing antibody has the potential to be far more efficient and cost-effective compared to discovery campaigns reliant on *in vivo* affinity maturation by successive vaccination and/or infection. In patient samples, broadly neutralizing antibodies must be identified from a large background of non-neutralizing anti-spike protein antibodies, while affinity maturation of a neutralizing antibody allows for fine tuning around a known neutralizing epitope. As in this study, filters can be included during phage panning, including heat treatment to remove unstable clones [14] and negative subtraction on BVP to remove polyreactive clones, to ensure that the final resulting candidates retain good developability profiles. This approach can be adapted for future therapeutic mAb development against SARS-CoV-2 or other viruses, even to allow for rapid development of broadly neutralizing therapeutic mAbs in real time.

## Supplementary Material

Supplementary_Data_Clean_tbad006

## Data Availability

The datasets used and/or analyzed during the current study are available from the corresponding author on reasonable request.

## ANIMAL RESEARCH STATEMENT

Rabbit immunization was performed by ProSci (San Diego, CA), following all Institutional Animal Care and Use Committee guidelines. Care was taken throughout the study to prevent any undue animal suffering.
