# Supplementary material for: Rapid engineering of SARS-CoV-2 therapeutic antibodies to increase breadth of neutralization including BQ.1.1, CA.3.1, CH.1.1, XBB.1.16, and XBB.1.5
Source: Antib Ther. 2023 Apr 13;6(2):108–18. doi: 10.1093/abt/tbad006 (PMC10262839; doi:10.1093/abt/tbad006)
Supplement: Supplementary_Data_Clean_tbad006 [file supplementary_data_clean_tbad006.docx]

# SUPPLEMENTARY DATA


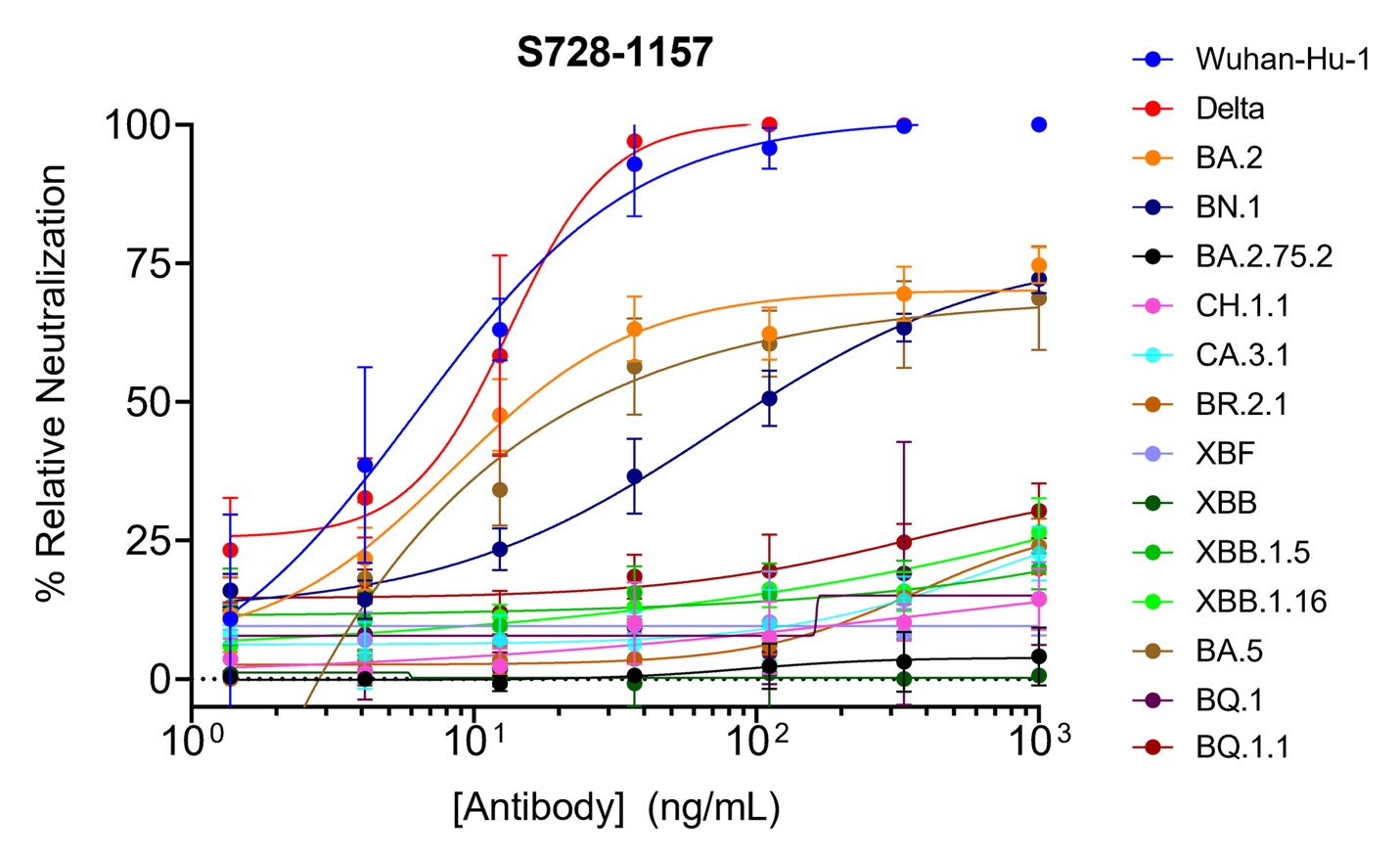


**Figure S1. sVNT of S728-1157 mAb.** S728-1157 showed potent neutralization of Wuhan-Hu-1 and Delta but reduced neutralization of BA.2, BA.5, and BN.1. No significant neutralization was observed for BA.2.75.2, BQ.1, BQ.1.1, XBB, XBB.1.5, XBB.1.16, CH.1.1, CA.3.1, XBF, or BR.2.1.


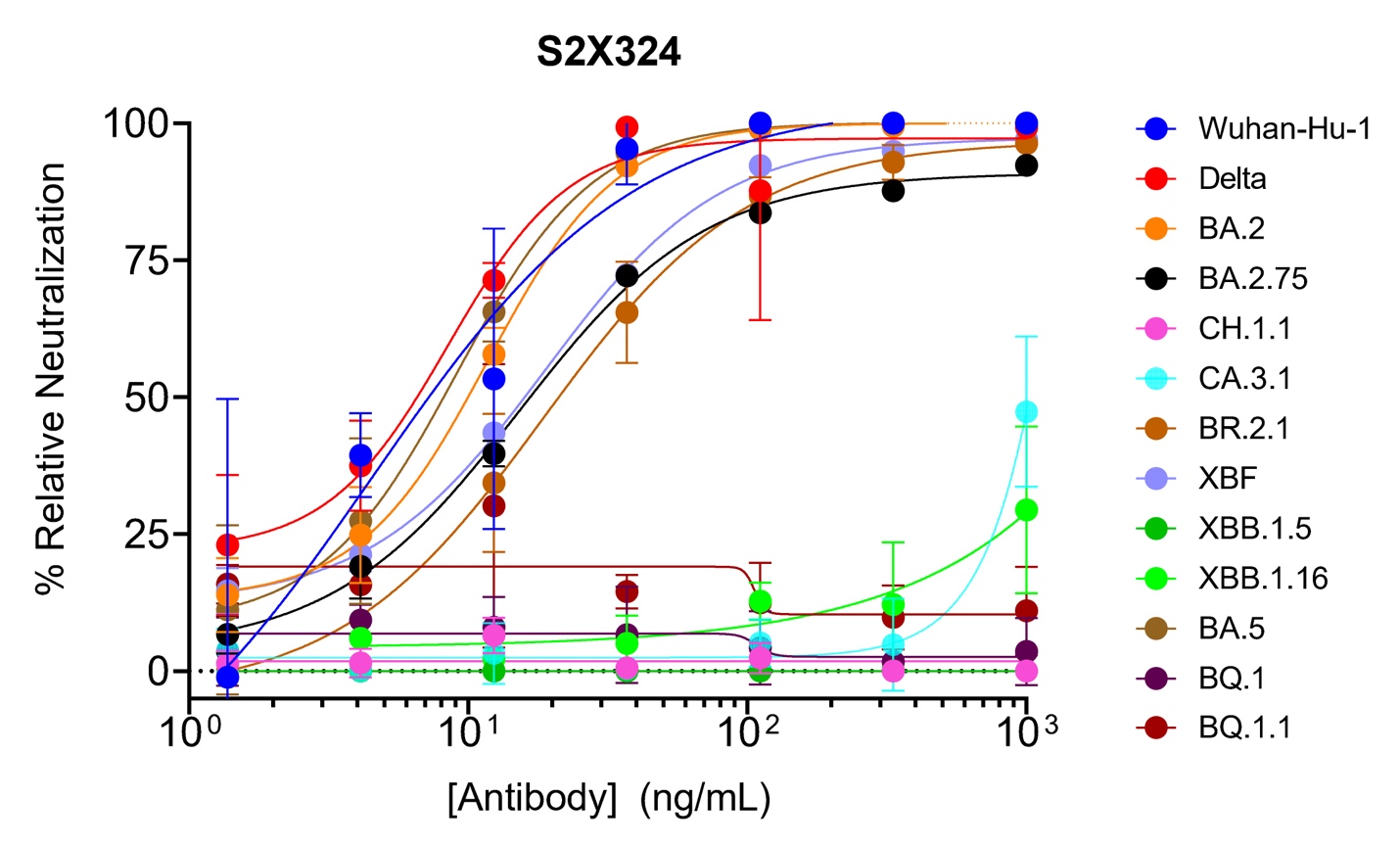


**Figure S2. sVNT of S2X324 mAb.** S2X324 showed potent neutralization of Wuhan-Hu-1, Delta, BA.2, BA.2.75, BA.5, XBF, and BR.2.1. No significant neutralization was observed for BQ.1, BQ.1.1, XBB.1.5, XBB.1.16, CH.1.1, or CA.3.1.


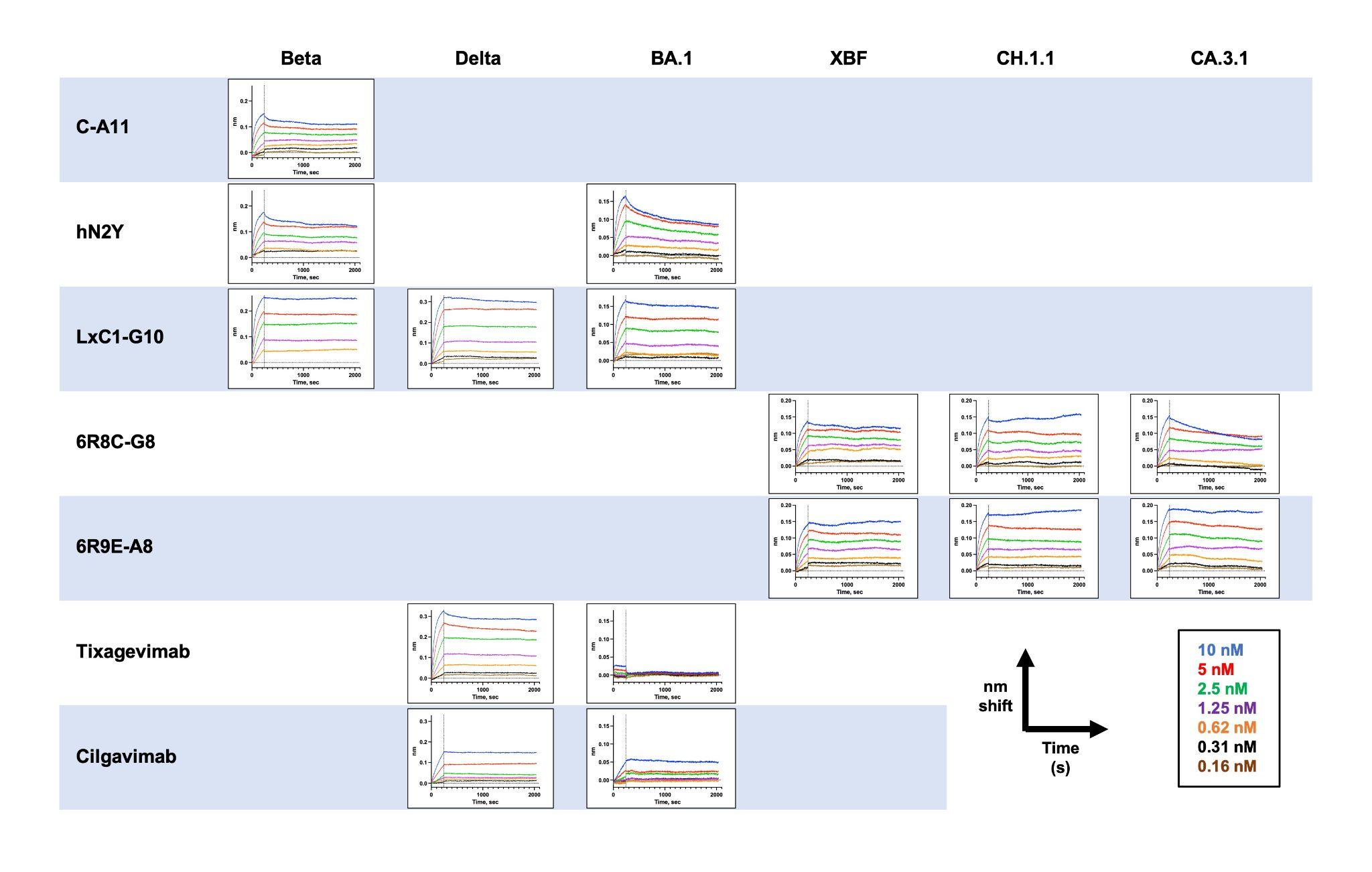


**Figure S3. BLI studies of mAbs on various spike trimer variants.** In each assay, biotinylated spike protein trimer was captured to the streptavidin-coated sensor surface and dipped into a 7-fold dilution series of IgG at a starting concentration of 10 nM. Binding was monitored for 4 minutes, followed by dissociation monitored for 30 minutes. Traces were referenced to a sensor omitting IgG.
